# Supplementary material for: Post-Transplant Cyclophosphamide Combined with Brilliant Blue G Reduces Graft-versus-Host Disease without Compromising Graft-versus-Leukaemia Immunity in Humanised Mice
Source: Int J Mol Sci. 2024 Feb 1;25(3):1775. doi: 10.3390/ijms25031775 (PMC10855770; doi:10.3390/ijms25031775)
Supplement: Supplementary file 1 [file ijms-25-01775-s001.zip › ijms-2771582-supplementary.pdf]

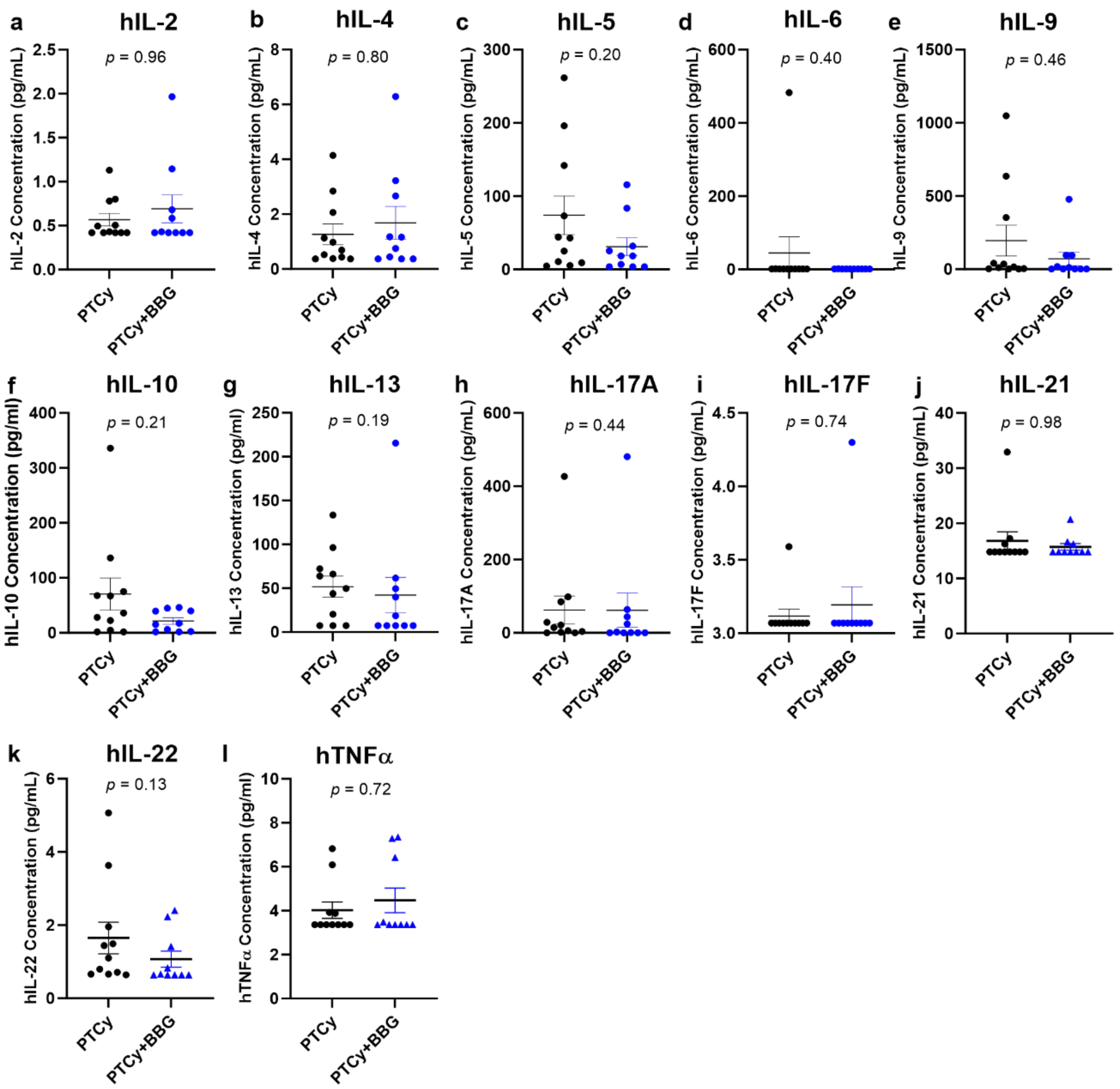

Figure S1. PTCy with BBG does not alter serum human cytokine concentrations compared to PTCy alone at endpoint**.** Sera from humanised mice treated with PTCy or PTCy+BBG (Figure 1) were examined for (**a**) human (h) IL-2, (**b**) hIL-4, (**c**) hIL-5, (**d**) hIL-6, (**e**) hIL-9, (**f**) hIL-10, (**g**) hIL-13, (**h**) hIL-17A, (**i**) hIL-17F, (**j**) hIL-21, (**k**) hIL-22 and (**i**) hTNF*α* by a flow cytometric LEGENDPlex kit. (**a-l**) Data presented as mean ± SEM. Symbols represent individual mice (*n* = 11, PTCy; *n* = 10, PTCy+BBG).

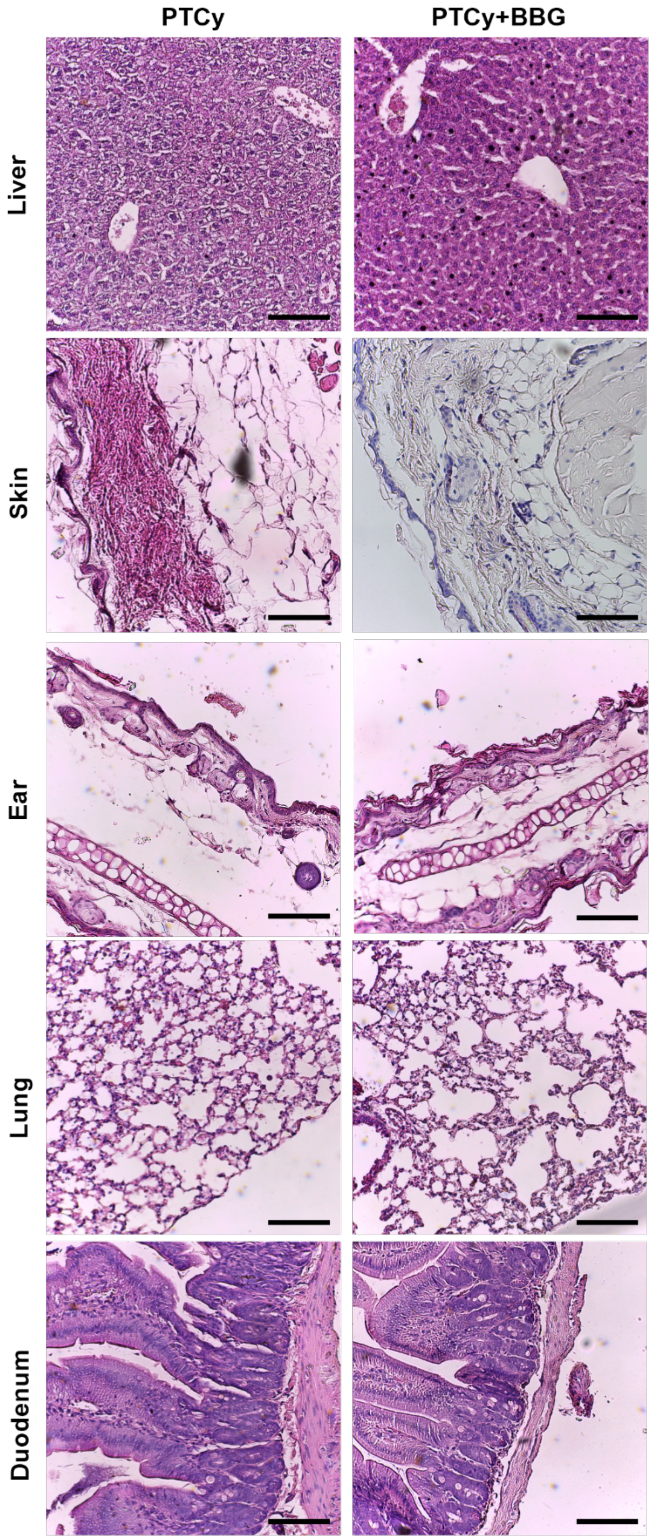

Figure S2. Histological organ GVHD was absent in humanised mice treated with PTCy and BBG or PTCy alone at Day 21. Haematoxylin and eosin-stained liver, skin, ear, lung and duodenum tissue sections from humanised mice treated with PTCy or PTCy+BBG (Figure 4) were examined for evidence of histological GVHD. Images representative of 5-8 mice per treatment group. Scale bars represent 100 μm.

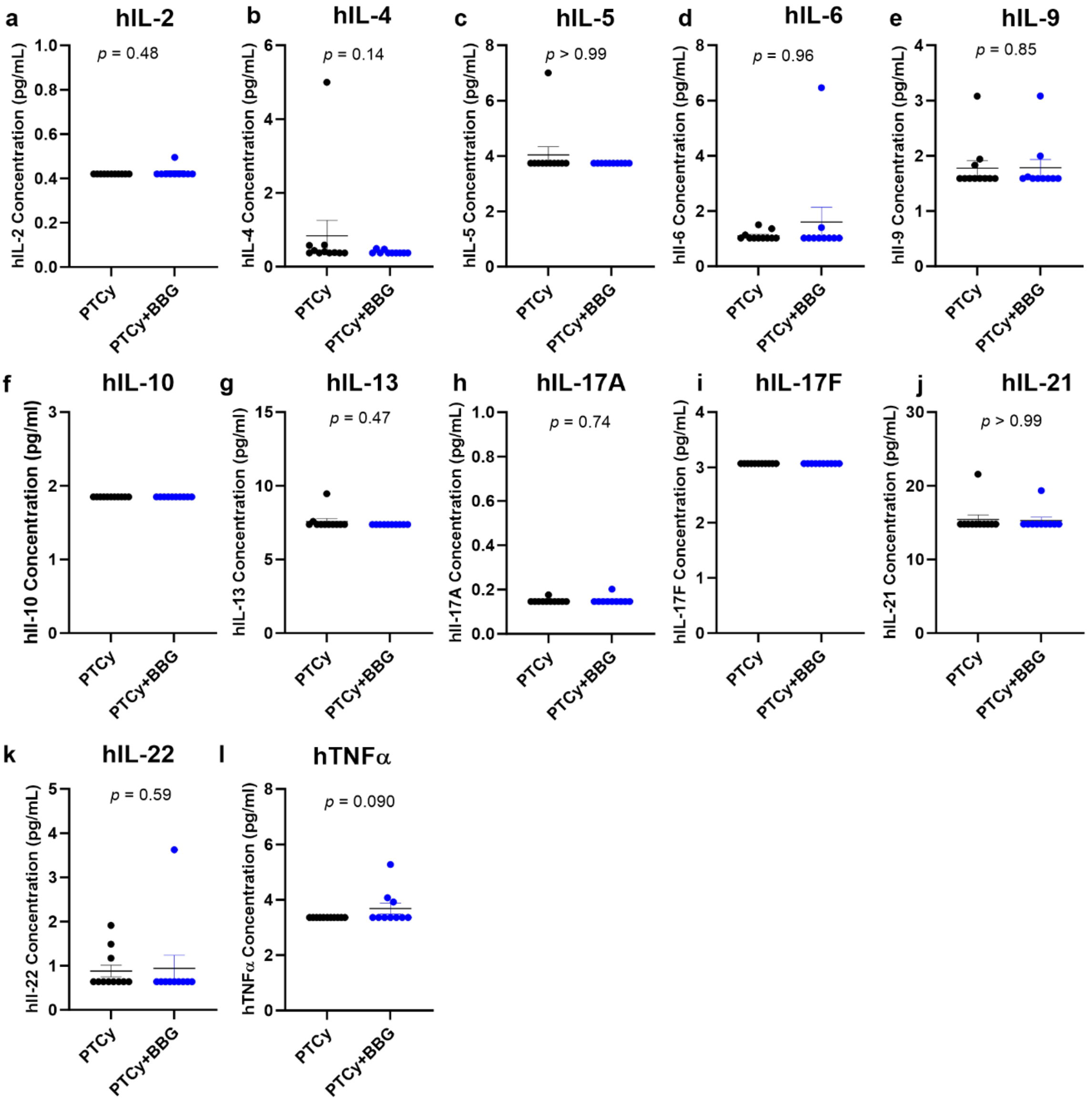

Figure S3. Human cytokines were mostly below the detection limit in humanised mice treated with PTCy and BBG or PTCy alone at Day 21. Sera from humanised mice treated with PTCy or PTCy+BBG (Figure 4) were examined for (**a**) human (h) IL-2, (**b**) hIL-4, (**c**) hIL-5, (**d**) hIL-6, (**e**) hIL-9, (**f**) hIL-10, (**g**) hIL-13, (**h**) hIL-17A, (**i**) hIL-17F, (**j**) hIL-21, (**k**) hIL-22, and (**m**) hTNFα by a flow cytometric LegendPlex kit. (**a-l**) Data presented as mean ± SEM. Symbols represent individual mice ($n = 11$, PTCy; $n = 10$, PTCy+BBG).
